# Supplementary material for: Evaluation of various methods of selection of B. subtilis strains capable of secreting surface-active compounds
Source: PLoS One. 2019 Nov 12;14(11):e0225108. doi: 10.1371/journal.pone.0225108 (PMC6850892; doi:10.1371/journal.pone.0225108)
Supplement: Attachment — Submitted filename: Response to Reviewers Plos One.docx [file pone.0225108.s001.docx]

**Detailed response to the reviewers' comments (PONE-D-19-24885)**

We would like to thank the Editor and the Reviewers for their comments and suggestions. We have revised the manuscript point by point according to the Reviewer’s comments. All suggested changes are marked in yellow in the revised text and described below.

We hope that the quality and readability of our manuscript has been improved.

**Additional Editor Comments (if provided):**

**In page-2, lines 39-40, after CMC give reference.**

The additional information has been added in line 40.

**Response to Reviewer No. 1 comments:**

**I consider this paper to be of interest to the PLOS One communities, and I am receptive towards publication subject to a minor comment. There has actually been prior work on computing peptides to understand surfactins, for example:**

**Biophysical Journal 85, 1377-1391 (2003)**

**Physical Chemistry Chemical Physics, 20, 18158-18168 (2018)**

**In principle, computation could also be used to complement the experiments investigated in this manuscript. I am not asking the authors to carry out any calculations; however, it should probably be mentioned that such studies could complement the experiments discussed in this work.**

The additional information has been added in lines 338-345, 566-570 according to the Reviewer suggestion.

**Response to Reviewer No. 2 comments:**

**1. How did you determine that strains were different? Also, can you provide any additional information about these strains (e.g. 16S)?**

The additional information about *B. subtilis* strain identification has been added in Isolation of *Bacillus subtilis natto* strains section (lines 128-130) according to the Reviewer suggestion.

The isolated *B. subtilis* strains exhibited different metabolic features (a different ability to perform hemolysis, reduce surface tension and produce surfactin), which was also confirmed by MLST (multilocus sequence typing) analysis. Strains capable of producing the highest amount of surfactin (BS15, BS19, BS24, BS43) showed allele sequence variability in the *pta* (phosphate acetyltransferase) locus.

**2. No information is provided regarding submerged culture. Please provide more details.**

All SmF parameters i.e. composition of the medium, culture time, pH, inoculum size have been added in Submerged fermentation (SmF) conditions section (lines 131-141).

**3. The correlation between surfactin production and surface tension is not too strong. I wonder if a multivariate regression involving the different isoforms would yield a better correlation?**

The main aim of our work was to develop a method that would allow an effective and possibly fast selection of strains capable of surfactin biosynthesis. The use of the degree of surface tension reduction of the culture medium as one of the selection criteria for potential surfactin producers has significantly reduced the screening time. To this end, we determined the concentration of surfactin produced by strains that reduced surface tension by 33-35%. The value of the correlation coefficient between the percentage reduction in surface tension during the culture and the concentration of surfactin produced, which was about 0.5, indicated the existence of a relationship between these two variables. This confirms the correct choice of method for selecting surfactin producing strains. On this basis, we also conclude that after examining the other 24 strains for surfactin biosynthesis, the correlation coefficient value would increase. On the other hand, multiple regression does not seem to be the appropriate statistical tool for this study. For the estimators calculated by the method of least squares to exist and have the desired properties (estimators should be unbiased and efficient), certain assumptions must be met. One of them is the lack of collinearity of independent variables (concentrations of surfactin isoforms). In this study, the value of the correlation coefficient between independent variables is much higher than that between independent variables and the dependent variable, i.e. the percentage of surface tension decrease. In the case of D, E, F, C isoforms it is even ≥ 0.9. The use of a multiple regression model, explaining the variability of surface tension reduction as a result of biosynthesis of individual surfactin isoforms, would require enlarging the data set (which was beyond the scope of the work) and perhaps removing the most strongly collinear variables, i.e. catalysts.
